# Evaluating a transdisciplinary supportive care model for advanced liver disease: Results of the *Liver Life* pilot randomized controlled trial (RCT)

**DOI:** 10.1017/S1478951526101795

**Published:** 2026-02-27

**Authors:** Sarah Pullen, Tazeen Majeed, Sarah Russo, Mary-Anne Dieckmann, Christopher Oldmeadow, Olivia Wynne, Caroline Kuhne, Jane Kerr, John Attia, Katie Wynne

**Affiliations:** 1 Hunter New England (HNE) Health, Supportive Care for Chronic Disease, John Hunter Hospital, New Lambton Heights, NSW, Australia; 2 Equity in Health and Wellbeing Program, Hunter Medical Research Institute (HMRI), New Lambton Heights, NSW, Australia; 3 School of Medicine and Public Health, University of Newcastle, New Lambton Heights, NSW, Australia; 4 Hunter New England (HNE) Health, Department of Nephrology, John Hunter Hospital, New Lambton Heights, NSW, Australia; 5 Hunter New England (HNE) Health, Department of Palliative Care, Tamworth Rural Referral Hospital, Tamworth, NSW, Australia; 6 Hunter Medical Research Institute (HMRI), New Lambton Heights, NSW, Australia; 7 Hunter New England (HNE) Health, Chronic Disease Network, Tamworth, NSW, Australia; 8 Department of Medicine, New Lambton Heights, NSW, Australia; 9 Hunter New England (HNE) Health, Diabetes & Endocrinology, New Lambton Heights, NSW, Australia

**Keywords:** Advanced liver disease, Palliative care, randomized controlled trial, transdisciplinary, supportive care, Health service utilisation

## Abstract

**Objectives:**

Patients with advanced liver disease (ALD) may benefit from the early integration of supportive care toward the end of life. Engagement with supportive and palliative care could decrease disease-related distress and alleviate pressure on the health system. This trial evaluated whether a transdisciplinary supportive care model, aligned with standard care and guided by patient- and carer-identified needs, could optimize health service utilization and outcomes for patients and carers living with ALD.

**Methods:**

A 90-day multicenter, mixed-methods pilot randomized controlled trial, *“Liver Life,”* was conducted at 1 regional tertiary and 1 rural referral hospital in NSW, Australia. The intervention group received patient- and carer-centered supportive care interventions during 5 scheduled allied health-led outpatient visits, alongside ongoing standard care. This paper reports health service utilization and associated costs, and participant-reported measures.

**Results:**

Over 90 days, emergency department presentations were reduced by 66% (incidence rate ratio: 0.34 [0.13–0.80]), and hospital admissions by 64% (incidence rate ratio: 0.36 [0.12–0.98]). Intervention patients were 5 times more likely to have more days “alive and out of hospital” than those receiving standard care alone (odds ratio: 5.34 [1.43–22.1]). As a result, the overall cost of health service use per intervention patient was less than half that of standard care alone.

**Significance of results:**

The *Liver Life* trial demonstrated the feasibility, acceptability, efficacy, and potential cost savings of a transdisciplinary supportive care model for ALD patients and their caregivers. Future research should investigate the sustainability and transferability of this approach to other populations and other chronic diseases.

## Graphical abstract

**Figure:**
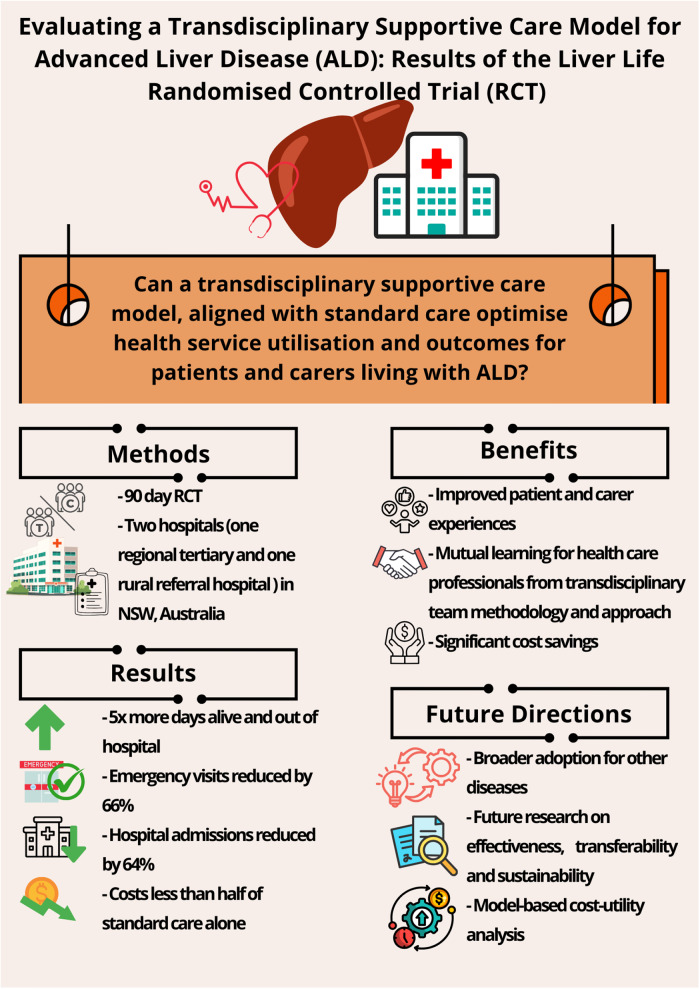

## Introduction

Advanced liver disease (ALD) is an increasingly pressing public health problem (Kim et al. 2022). It is estimated that 1.5 billion people globally are living with chronic liver disease, with the prevalence increasing in parallel with the etiological factors of obesity and alcohol consumption (Moon et al. 2020; Cheemerla and Balakrishnan 2021). ALD is often underdiagnosed in primary care settings, leading to hospital presentations in advanced stages, thereby minimizing the opportunities for effective early intervention (El-Atem et al. 2016, 2017; Jordan and Tandon 2020). The prognosis for people with ALD with a Child-Pugh Score of B or C is typically less than 2 years, with curative options remaining largely unavailable (Haj and Rockey 2018). Up to 50% of ALD patients assessed for liver transplantation are deemed ineligible due to their comorbidities, psychosocial issues, or the presence of progressive hepatic cancer (Mazzarelli et al. 2018). Among those deemed ineligible for transplantation, only one-fifth receive palliative care input, which typically occurs in a hospital setting during the last few days of life (Verma et al. 2019).

ALD patients experience a high symptom burden, with common symptoms such as pain, breathlessness, muscle cramps, sleep disturbances, and psychosocial distress significantly impairing quality of life (Peng et al. 2019; Moon et al. 2020; Naik et al. 2020; Valery et al. 2023). Health information is frequently delivered at a level exceeding health literacy, and patients often express a need for more practical assistance and psychosocial support (Low et al. 2018). Poor health literacy, inadequate disease-related knowledge, and unmet care needs have been demonstrated to correlate with increased unplanned healthcare service utilization and expenditure (Peng et al. 2019; Moon et al. 2020; Naik et al. 2020; Valery et al. 2023). Additionally, advance care planning (ACP) in ALD is often inadequate or neglected prior to death (Plunkett et al. 2019; Sprange et al. 2019). In Australia, approximately 6 million people experience liver disease, resulting in an estimated societal cost of $51 billion annually (El-Atem et al. 2016).

Recent guidelines from the American Liver Disease Committee advocate for the early introduction (at the time of diagnosis) of a palliative care approach involving a multidisciplinary team with palliative care input (Rogal et al. 2022). Key considerations include improved communication regarding care goals and the provision of symptomatic and psychological support (Kim et al. 2022). Supportive care encompasses the core palliative care principles of complex symptom management, education for patients and carers, psychosocial, emotional, spiritual, and functional care, with coordination of care services and ACP. This support is offered alongside ongoing active medical therapy (Woodland et al. 2020).

Despite growing recognition of the need for supportive care in ALD, there remains a significant gap in evidence regarding the effectiveness of structured, patient-centered interventions in this population. Most existing studies are retrospective, descriptive, or focused on end-of-life care, with limited prospective data evaluating the impact of timely supportive care on health service utilization, patient-reported outcomes, or cost-effectiveness (Patel and Ufere 2020; Wright et al. 2022). Evidence highlights the delay or absence of referral to palliative care. A retrospective study reviewing 166 eligible ALD patients for palliative care referral found that 25 were referred for services, all of whom died within 3 weeks of referral (Chen et al. 2021). Although a US study demonstrated an increase in palliative care referrals between 2006 and 2012, fewer than 1 in 15 ALD patients received such care despite poor prognosis (Rush et al. 2017; Shinall et al. 2022). This lack of appropriate care at the end of life means that ALD patients may spend their final year undergoing invasive therapies, with repeated hospital admissions, and unmet psychosocial and emotional needs prior to death.

While supportive care models have been successfully implemented in malignant conditions and other chronic disease contexts such as renal failure, their application in ALD remains underexplored and inconsistently adopted in clinical practice. The successful state-wide implementation of Kidney Supportive Care in NSW, Australia, has demonstrated that a supportive care model can be scaled to effectively address patient-identified needs while filling crucial gaps in clinical practice for people living with chronic diseases (Siriwardana et al. 2020). However, no prospective randomized controlled trial (RCT) to date has evaluated a structured, transdisciplinary supportive care model tailored to the needs of ALD patients and their carers. This trial addresses this critical evidence gap by exploring how the foundational principles of supportive care can be effectively integrated into a model of care for patients with ALD, and whether such a model can improve health outcomes, reduce unplanned healthcare use, and deliver economic value. The findings have the potential to inform future models of care and health policy for this high-need, underserved population.

## Methods

### Study setting and participants

Participants and their carers were recruited from 1 regional tertiary (Site 1: John Hunter Hospital, Newcastle, NSW) and 1 rural referral hospital (Site 2: Tamworth Rural Referral Hospital, Tamworth, NSW) within a single local health district (LHD). Adult patients with ALD classified as Child-Pugh Score B or C were recruited from inpatient and outpatient settings. Patients with or without a suspected or diagnosed hepatocellular carcinoma, and those awaiting liver transplantation, were eligible for the study. Patients who were post-transplantation or had previous or current engagement with palliative care services were excluded. Ethical approval was provided by the Hunter New England Ethics Committee [2020/ETH02259].

### Randomization and data management

Trial data were collected and managed using the REDCap electronic data capture tools hosted within Hunter New England LHD (Hunter Medical Research Institute 2022). Participants were randomized via REDCap in a 1:1 ratio to either the intervention or standard care group, stratified by trial site and Child-Pugh score (B or C). Semi-structured interviews with patients, carers, and clinicians were performed before and at the conclusion of the trial; this paper will focus exclusively on the quantitative component. This focus enables a rigorous evaluation of the intervention’s efficacy through objective, reproducible metrics essential for informing clinical decision-making and policy development in a high-stakes healthcare context.

### Intervention

Supplementary Figure 1 presents the framework for the supportive care model. Over the 90-day intervention, 5 additional allied health outpatient visits of approximately 45 minutes each were conducted as a group consultation with all members of the supportive care multidisciplinary team, in addition to standard care. The supportive care multidisciplinary team comprised a nurse, social worker, and dietitian from the existing hepatology team. Importantly, all clinicians came together with intervention participants in the same physical or virtual space during supportive care consultations. Participants attended visits at baseline, Week 4, Week 8, and Week 12. Carers attended 3 visits: with the person they care for at baseline and Week 12, and individually at Week 6. Palliative care support from a consultant, advanced trainee, or nurse practitioner, and input from an Aboriginal health worker or liaison officer were sought as appropriate. Consultations were delivered in-person or virtually, depending on participant needs.

Standard care for control and intervention participants was the same, conducted in multidisciplinary outpatient clinic settings. Frequency of outpatient appointments at both sites was determined according to individual patient need, with appointments on average occurring every 6 months. At Site 1, a 15-minute standard care outpatient appointment was held with either a Gastroenterologist (*n* = 1), Hepatologist (*n* = 1), Gastroenterology registrar (*n* = 1), or Hepatology Nurse Practitioner (*n* = 1) as the primary clinician. The primary clinician was allocated by the outpatient nurse coordinator on the day of the appointment, taking into consideration the patient’s history, previous clinical consultations, and clinician availability. After review with the primary clinician, the patient had access to a dietitian (*n* = 1) and a social worker (*n* = 1). Appointments with these clinicians were either planned or directed by the primary clinician on the day of the appointment, pending patient need. At Site 2, participants attended the outpatient appointment with a General Physician with gastroenterology training (*n* = 2) as the primary clinician. The primary clinician was allocated by the outpatient nurse coordinator on the day of the appointment with consideration of patient history, previous clinical consultations, and clinician availability. After review with the primary clinician, the patient had access to a dietitian (*n* = 1) and a clinical nurse consultant (*n* = 1). Appointments with these clinicians were either planned or directed by the primary clinician on the day of the appointment, pending patient need.

### Data collection and clinical triggers for intervention

Participant-reported measures (Table 1) were completed on a handheld electronic device within 48 hours prior to each supportive care consultation. Pre-determined clinical triggers for intervention (Supplementary files, Table 1) were established prior to trial commencement in consultation with local clinical experts and were embedded in the REDCap database. These clinical triggers for intervention highlighted any participant-reported symptom or concern that elicited moderate or higher levels of distress or discomfort and prompted action from specific clinical roles within the supportive care multidisciplinary team. On completion of participant-reported measures, the REDCap database generated an automated summary report of measure outcomes, which was available to the clinical team in real time. The summary report and any voiced concerns of participants guided the content of each supportive care consultation. This aimed to facilitate participant-driven and timely symptom management, psychosocial care, or medical nutrition therapy, and reduce clinical inertia.

**Table 1.** Schedule of assessment measures for patients and carers

Visit Number	1	2	3	4	5
**Time**	Week 0	Week 4	Week 6	Week 8	Week 12
**Patient-reported measure (PRM)**					
Integrated Palliative Outcomes Scale (IPOS)	X	X		X	X
EuroQoL 5 Dimension 5 Level (EQ-5D-5L)	X	X		X	X
Malnutrition Screening Tool (MST) and Subjective Global Assessment (SGA)	X				X
**Carer-reported measure (CRM)**					
Carer Experience Scale (CES)	X		X		X
Carer Support Needs Assessment Tool (CSNAT)	X		X		X
**Semi-structured Interviews**	X				X

This table outlines the timing and type of assessments conducted across the 5 study visits (baseline to Week 12). Patient-reported measures include the Integrated Palliative Outcomes Scale (IPOS), EuroQoL 5-Dimension 5-Level (EQ-5D-5L), and nutritional assessments using the Malnutrition Screening Tool (MST) and Subjective Global Assessment (SGA). Carer-reported measures include the Carer Experience Scale (CES) and the Carer Support Needs Assessment Tool (CSNAT). Semi-structured interviews were conducted at selected time points to explore patient and carer experiences in greater depth.

Control and intervention group participants completed participant-reported measures at the same time points; however, there was no intervention for control participants.

A patient- or carer-initiated telephone support line facilitated “just-in-time” access to the hepatology nurse or allied health coordinator, allowing interventions to be triggered between scheduled appointments as required.

### Outcomes

The primary outcomes were: (1) number of emergency presentations; (2) number of unplanned hospital admissions; and (3) days alive and out of hospital, over the 90-day intervention period. These were selected to comprehensively assess the intervention’s impact on both patient-centered experiences (days alive and out of hospital) and health service utilization (emergency presentations and unplanned admissions). Data were collected from public hospital records throughout the LHD.

To explore the primary outcomes from another aspect, the parameter “days alive and out of hospital” was compared between intervention and control groups using the Wilcoxon rank sum test.

The secondary outcomes were:

- Evidence of ACP: documented plans and/or directives, resuscitation orders, and/or documented evidence of planning discussions verified from the clinical record.
- Symptom burden: Integrated Palliative Outcome Scale (IPOS) scores.
- Carer burden: Carer Experience Scale (CES) and Carer Support Needs Assessment Tool (CSNAT).
- Economic benefit: a cost–utility analysis across the 90-day study is currently in progress. This paper presents costs and Quality-Adjusted Life Years (QALYs) calculated from EuroQoL (EQ-5D-5L) scores.
- Feasibility and acceptability: semi-structured interviews with clinicians, patients, and carers (not discussed here).

### Statistical analysis

Data were extracted from REDCap and analyzed using SAS v9.4 (SAS Institute Inc 2025). Descriptive statistics for patient and carer characteristics were presented as counts (%) or means (SD) and medians (minimum, maximum). Analysis followed the intention-to-treat principle, analyzing all participants according to their allocated treatment arm.

Relative differences in count outcomes (90-day emergency presentations, number of unplanned hospital admissions, and number of unplanned clinical contacts for symptom relief) were estimated using a negative binomial regression model, with the log of the follow-up time as an offset (follow-up time being censored at date of death), and adjusting for treatment group and site, with exponentiated parameter estimates representing incidence rate ratios and 95% confidence intervals (CIs). For the outcome “days alive and out of hospital,” adjusted relative differences were assessed using an ordinal logistic regression model, due to the distribution of values being constrained by a 90-day ceiling defined by the duration of the study. Days alive and out of hospital (at 90 days) was compared between intervention and control groups using a proportional odds ordinal regression model (adjusting for treatment group and site), with the treatment effect presented as an ordinal odds ratio (reflecting the odds of a participant in the intervention group having a higher number of days alive and out of hospital than a participant in the control group) with 95% CI and *p*-value.

Linear mixed models were used to estimate differences in carer burden and quality of life between intervention and control groups at each follow-up time point. Models included fixed categorical effects for time, treatment group, and their interaction, along with the baseline value of the outcome variable. Random subject-level intercepts were included to account for repeated measures on the same participants over time. Least squares mean estimates (LS means) of the baseline-adjusted treatment effect were calculated at each time point, with 95% CIs. A significance level of *p* < 0.05 (2-tailed) was used to indicate statistical significance.

### Sample size

This study was primarily designed as a pilot RCT to assess the feasibility, acceptability, and preliminary efficacy of a transdisciplinary supportive care model for patients with ALD. Based on a review of service data from both sites, the research team anticipated recruiting approximately 50 participants across both sites (25 per arm). This sample size was estimated to provide ∼80% power at α = 0.05 to detect either a 40% reduction in unplanned clinical contact (e.g. from 50% down to 10%) or a difference of 0.8 standard deviations (Cohen’s *d* = 0.8) in patient-reported outcome measures.

### Healthcare utilization costs

Taking a health service perspective, the costing analysis included direct healthcare costs associated with the study’s primary outcomes of emergency department (ED) presentations, inpatient hospitalizations, and outpatient appointments over a 12-week time horizon.

Resource use was identified through linked administrative datasets supplied by the LHD data custodian after the requisite approvals. De-identified participant data were sourced from Emergency Department Data Collection and Admitted Patient Data Collection databases, and outpatient appointment information was sourced from the LHD Community Health database.

Hospitalizations, ED presentations, and outpatient appointments were costed using publicly available cost weights and the National Efficient Price (NEP) from the Independent Health and Aged Care Pricing Authority (IHACPA) . All costs were adjusted to 2024 Australian dollars using relevant healthcare inflation indices sourced from the Australian Bureau of Statistics (Australian Bureau of Statistics 2025).

### Quality-adjusted life years

QALYs were calculated using EQ-5D-5L utility scores (Herdman et al. 2011; Janssen et al. 2013) collected at weeks 0, 4, 8, and 12. Utility values were derived using the Australian EQ-5D-5L value set (Norman et al. 2023), which reflects population preferences for health states. Each health state was converted into a utility index score ranging from −0.30 (worst health state) to 1.00 (full health). Time was expressed in years, and QALYs were estimated by calculating the area under the utility curve using the trapezoidal rule, assuming linear interpolation between time points (Whitehead and Ali 2010). The total QALYs over the 12-week period were obtained by summing the QALYs across all intervals.

## Results

**Figure 1.**
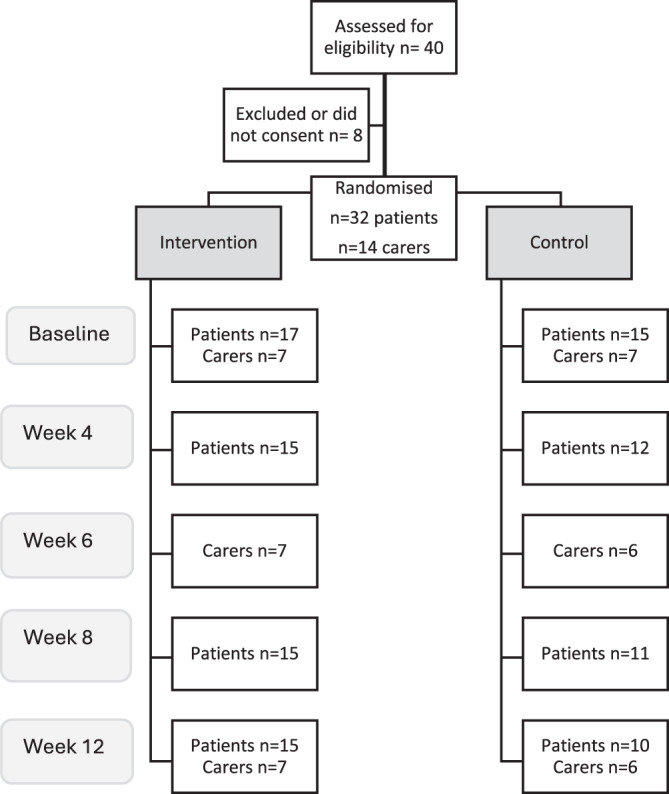
Participant flow and recruitment. This figure outlines the screening, randomization, and follow-up of patients and carers across the intervention and control groups. Of the 40 individuals assessed for eligibility, 32 patients and 14 carers consented and were randomized. The diagram shows the number of patients and carers contributing data at each study time point (baseline, Week 4, Week 6, Week 8, and Week 12) for both groups, illustrating attrition and patterns of participant engagement over the study period.

A total of 40 participants were assessed for eligibility, with 8 excluded (Figure 1). Thirty-two patients (Site 1: *n* = 24; Site 2: *n* = 8) and 14 carers (Site 1: *n* = 11; Site 2: *n* = 3) were randomized (Table 3). The mean age of participants was 57.7 years, with a mean body mass index of 27.7 kg/m^2^. Two-thirds of participants were male, most were unemployed, and lived with family, a friend, or others. A quarter of patients reported current alcohol use (see Table 2 for summary of patient characteristics at baseline). During the trial period, 1 patient died (with carer withdrawn, Site 1), 1 patient withdrew (Site 2), and 5 patients were lost to follow-up (Site 1: *n* = 2; Site 2: *n* = 3). In total, 25 patients and 13 carers completed the 90-day study; all intervention visits were completed by 95% (*n* = 19/20) of intervention participants (patients *n* = 12, carers *n* = 7). Over the course of the study, 25 patient participants triggered 92 interventions, and 13 carers triggered 13 interventions (Supplementary files, Table 1).

**Table 2.** Summary of patient characteristics at baseline

Variable	Control *N* = 15	Intervention *N* = 17	Total *N* = 32
Patients			
Site 1	11 (73%)	13 (76%)	24 (75%)
Site 2	4 (27%)	4 (24%)	8 (25%)
Gender			
Male	13 (87%)	8 (47%)	21 (66%)
Female	2 (13%)	9 (53%)	11 (34%)
Age			57.7 (10.3)
Mean (SD)	59.3 (10.4)	56.2 (10.2)	
Median (IQR)	59 (50–64)	54 (52–62)	
Range	48–87	35–81	
Height mean (SD)	1.8 (0.11)	1.7 (0.07)	1.7 (0.10)
Weight mean (SD)	89.4 (20.6)	76.3 (17.2)	82.4 (19.7)
BMI mean (SD)	29.0 (7.3)	26.6 (5.4)	27.7 (6.4)
HCC diagnosis			
Site 1	3 (37.5%)	2 (15.3%)	5 (15.6%)
Site 2	0 (0%)	0 (0%)	0 (0%)
Transplant evaluation			
Site 1	0 (0%)	2 (11.8%)	2 (6.3%)
Site 2	0 (0%)	0 (0%)	0 (0%)
Living arrangements			
Lives alone	0 (0%)	5 (29%)	5 (16%)
Lives with another	15 (100%)	12 (71%)	27 (84%)
Employment status			
Unemployed	6 (40%)	14 (82%)	20 (63%)
Casual	0 (0%)	0 (0%)	0 (0%)
Part-time	1 (6.7%)	1 (5.9%)	3 (0%)
Full-time	3 (20%)	0 (0%)	3 (9.4%)
Study	0 (0%)	0 (0%)	0 (0%)
Volunteer work	0 (0%)	0 (0%)	0 (0%)
Retired	5 (33%)	2 (12%)	7 (21.8%)
Alcohol			
Current	4 (27%)	4 (24%)	8 (25.0%)
Previous use	8 (53%)	12 (71%)	12 (62.5%)
Never	3 (20%)	1 (5.9%)	1 (12.5%)
Smoking			
Current	4 (27%)	8 (50%)	12 (37.5%)
Previous use	5 (33%)	5 (31%)	10 (31.3%)
Never	6 (40%)	2 (12%)	8 (25.0%)
Prefer not to disclose	0 (0%)	1 (6.2%)	1 (3.1%)
Drug use			
Current	2 (13%)	2 (12%)	4 (12.5%)
Previous use	1 (6.7%)	5 (29%)	6 (18.8%)
Never	11 (73%)	7 (41%)	18 (56.2%)
Prefer not to disclose	1 (6.7%)	3 (18%)	4 (12.5%)
Highest education			
Primary	1 (6.7%)	0 (0%)	1 (3.1%)
High school (to year 10)	5 (33%)	10 (59%)	15 (46.9%)
High school (to year 12)	0 (0%)	2 (12%)	2 (6.3%)
TAFE	0 (0%)	3 (18%)	3 (9.4%)
Apprenticeship	3 (20%)	1 (5.9%)	4 (12.5%)
Tertiary	6 (40%)	1 (5.9%)	7 (21.9%)
First nations			
Yes	0 (0%)	3 (18%)	3 (9.4%)
No	15 (100%)	14 (82%)	29 (91%)
Regular GP			
Yes	15 (100%)	15 (88%)	30 (93.7%)
No	0 (0%)	2 (12%)	2 (6.3%)

This table summarizes demographic, clinical, and social characteristics of participants at study enrollment. Variables include site of recruitment, gender, age, anthropometric measures, hepatocellular carcinoma (HCC) diagnosis, transplant evaluation status, living arrangements, employment status, alcohol and smoking history, drug use, highest education level, First nations identification, and regular general practitioner (GP) access. Values are presented as counts and percentages, with means (SD), medians (IQR), and ranges reported where appropriate.

### Impact on healthcare usage

The average ED presentations within 90 days were higher in the control group (mean: 1.40, SD: 1.12) compared with the intervention group (mean: 0.47, SD: 0.80). Over the 90-day period, the intervention group experienced a 66% reduction in ED presentations of any cause (incidence rate ratio: 0.34 [0.13–0.80]), along with a 64% reduction in hospital admissions of any cause (incidence rate ratio: 0.36 [0.12–0.98]) compared to the control group (Table 3). As expected, outpatient contacts increased due to the intervention design. No significant differences in the relative benefit of the primary outcomes were noted within the intervention group between participants receiving in-person visits versus virtual care (data not shown). The number of actions triggered from each patient/carer-reported measure was similar across both modalities of delivery.

**Table 3.** Outcome variable summaries by treatment group

(a) The mean and standard deviations for the number of admissions, presentations, and outpatient occasions of service are shown for the control and intervention groups				
Variable	Control *N* = 15	Intervention *N* = 17	IRR or OR (95% CI)	*p*-value^a^
**Number of ED presentations (at 90 days)**			IRR 0.34 (0.13–0.80)	0.007
Mean (SD)	1.4 (1.1)	0.5 (0.8)		
Median (IQR)	1.0 (1.0–2.0)	0.0 (0.0–1.0)		
Range	0.0–4.0	0.0–3.0		
**Number of admissions (at 90 days)**			IRR 0.36 (0.12–0.98)	0.041
Mean (SD)	0.8 (0.8)	0.3 (0.6)		
Median (IQR)	1.0 (0.0–1.0)	0.0 (0.0–0.0)		
Range	0.0–2.0	0.0–2.0		
**Number of outpatient services (at 90 days)**			IRR 2.03 (1.29–3.23)	0.024
Mean	3.4 (2.5)	7.9 (5.6)		
Median	2.0 (2.0–5.0)	7.0 (3.0–13.0)		
Range	0.00–2.00	0.0–17.0		
**Days alive and out of hospital (at 90 days)**			OR 5.34 (1.43–22.1)	0.021
Mean	80 (20)	88 (4)		
Median	87 (82–90)	90 (90–90)		
Range	12–90	75–90		
**Number of ED presentations (at 90 days)**			–	–
0	3 (20%)	11 (65%)		
1	6 (40%)	5 (29%)		
2	4 (27%)	0 (0%)		
3	1 (6.7%)	1 (5.9%)		
4	1 (6.7%)	0 (0%)		
**Number of admissions at 90 days (at 90 days)**			–	–
0	6 (40%)	13 (76%)		
1	6 (40%)	3 (18%)		
2	3 (20%)	1 (5.9%)		

(b) The relative differences are shown as regression results with IRRs for ED presentations and admissions				
	ED presentations	Admissions	Outpatient occasions	
Characteristic	IRR (95% CI), *p*-value	IRR (95% CI), *p*-value	IRR (95% CI), *p*-value	
Treatment factor				
Control	–	–	–	
Intervention	0.34 (0.13–0.8), 0.025	0.36 (0.12*–*0.98), 0.057	2.03 (1.29−3.23), 0.002	

This table presents descriptive and comparative outcomes at 90 days for the control and intervention groups, including emergency department (ED) presentations, hospital admissions, outpatient service use, and days alive and out of hospital. Means, medians (IQR), and ranges are reported alongside IRRs and odds ratios (ORs) with 95% CIs for between-group comparisons. Panel (a) summarizes raw outcome distributions and associated group differences (Wilcoxon rank-sum test). Panel (b) reports regression-derived IRRs for ED presentations, admissions, and outpatient occasions of service, adjusted for treatment group.

IRR = incidence rate ratio, CI = confidence interval.

a Wilcoxon rank-sum test.

### Patient outcomes

The median number of “days alive and out of hospital” was 90 days for the intervention group and 87 days for the control group, with the intervention group being 5 times more likely to have a higher number of days alive and out of hospital than the control group (odds ratio: 5.34 [1.43–22.1]).

ACP had been commenced by only 6% (*n* = 2/32) of all patient participants at the start of the study. At trial completion, the control group reported no additional instances of ACP discussions or documentation (*n* = 0/15), whereas 65% of participants in the intervention group (*n* = 11/17), engaged in ACP discussions or completed ACP documentation.

### Healthcare utilization costs

Supplementary Table 2 shows the estimated cost per visit to health services use based on the averages reported in the study. Table 4 shows the average number of visits to health services per patient and the subsequent estimated cost per patient for the trial follow-up period.

**Table 4.** Per patient average health service use and cost

	Average per patient			Average cost per patient ($)^a^			
	ED presentations	Hospital admissions	Outpatient appts	ED presentations	Hospital admissions	Outpatient appts	Total
Standard care (*n* = 15)	1.4	0.8	3.4	1,541	8,062	1,102	10,704
Intervention (*n* = 17)	0.5	0.3	7.9	557	1,832	2,560	4,948

This table summarizes mean per-patient use of emergency department (ED) presentations, hospital admissions, and outpatient appointments, together with the corresponding average costs (rounded to whole dollars). Cost comparisons between the standard care group and the intervention group illustrate differences in health service utilization over the study period.

a Costs are presented in whole dollars using commas as thousands separators.

### QALYs

Supplementary Table 3 presents descriptive statistics for QALYs, calculated using EQ-5D-5L scores reported by patients. Over the 12-week intervention period, participants in the intervention group accrued a mean of 0.18 QALYs (SD: 0.02), compared to 0.14 QALYs (SD: 0.06) in the standard care group. These results suggest a modest improvement in health-related quality of life among those receiving the intervention.

## Discussion

The *Liver Life* RCT demonstrated that a structured supportive care intervention significantly reduced unplanned hospital presentations and increased the number of days patients with ALD remained alive and out of hospital. Although no significant changes were observed in subjective symptom burden or self-reported carer burden, the care delivery from the supportive care multidisciplinary team effectively prevented emergency presentations. Utilizing participant-reported measures to trigger protocolized interventions is a technology-enabled concept aimed at negating the impact of clinician bias and overcoming clinical inertia in care planning and delivery, ensuring that care is tailored to the expressed needs of patients and their carers.

The importance of addressing carer needs should not be underappreciated. A recent literature review of palliative care in ALD highlighted the paucity of studies including caregiver outcomes, as well as the lack of RCTs (Mudumbi et al. 2018). This study provides novel evidence demonstrating that supportive care in ALD is both acceptable and efficacious. The existing literature consists of exploratory studies that investigate the integration of palliative aspects of care into chronic disease management to enhance consumer-driven care planning, postulating that this may achieve health outcomes that align with individual values and goals (Low et al. 2017; Valery et al. 2017; Plunkett et al. 2019; Naik et al. 2020). A UK feasibility trial involving a supportive care nurse showed promise in improving care coordination, ACP, and quality of life for patients with ALD and their carers, while reducing unplanned hospital admissions, outpatient non-attendance, and improving quality of life (Kimbell et al. 2018). However, the *Liver Life* trial is the first RCT to demonstrate the short-term benefits of an allied health-led supportive care model in ALD, with potential cost savings to the health service. The virtual mode of delivery of the intervention is particularly noteworthy. It provides access to specialist allied health, palliative care, and chronic disease services for those living in rural or remote areas, or for patients with high symptom burden, restricted mobility, and limited transport options (Muftah et al. 2023). Approximately one-third of *Liver Life* intervention participants attended the majority of their intervention visits using telehealth, incorporating a combination of telephone calls and a video platform, *My Virtual Care,* used by the health district. This may explain the high fidelity of the intervention and few episodes of failure to attend. Importantly, the intervention was equally effective across both delivery modalities, with no difference in triggering actions or health service utilization.

The coming together of all clinicians with patients and carers during consultations aided transdisciplinary experiential learning for clinicians. The transdisciplinary approach was successful in enhancing the clinicians’ scope of practice, professional networking, value for the contribution of diverse disciplines, and promoting collaboration and patient-centeredness in care planning and delivery.

The intervention resulted in greater participation in ACP, although accessing completed ACP documents by health professionals, whether in an emergency or non-emergency situation, remains challenging across NSW. Identification of the evidence of ACP in this study was impeded by inconsistent documentation and storage processes. Documents may be stored in paper or electronic databases of the LHD, or held by the patient, carer, or family in place of residence, or by external agencies such as family law practices. Currently, there is no statewide policy document providing guidance on storage and easy access to available ACP documents, with the exception of access to *My Health Record,* which requires patient consent (NSW Health 2019). A more consistent process would assist clinicians in identifying this gap in care and utilizing documents effectively in clinical practice.

Total health service costs were higher in the standard care group, primarily driven by a greater number of hospital admissions and a higher cost per admission. Although outpatient costs were higher in the intervention group due to more frequent visits, this increase was offset by the substantial reduction in hospital admissions. As a result, the overall cost per patient in the intervention group was less than half that of standard care alone. Given the advanced nature of liver disease in this cohort, no significant change in participants’ self-reported symptom burden (IPOS), or in self-reported carer burden (CSNAT), or CES was expected. However, despite the ongoing progression of the advanced and terminal condition, QALYs were marginally higher in the intervention group. This suggests an improvement in health-related quality of life due to the intervention. These findings are supported by systematic review evidence indicating the effectiveness of integrated care interventions in improving patient quality of life for patients with chronic conditions (Flanagan et al. 2017). These findings highlight the need for further research to explore alternative approaches or modifications to the current intervention that might more effectively address symptom and carer burdens.

### Strengths and limitations

This study has several notable strengths. It is the first RCT to evaluate a transdisciplinary supportive care model in patients with ALD and their carers, addressing a critical gap in the literature. The trial was conducted across both a regional tertiary and a rural referral hospital, enhancing the generalizability of findings to diverse healthcare settings. The intervention design was technology-enabled and patient-centered, using participant-reported outcome measures to trigger protocolized care actions – an innovative approach that mitigates clinician bias and overcomes clinical inertia. The inclusion of carers as participants is another strength, contributing novel insights into caregiver needs and experiences, which are often underrepresented in ALD research. The hybrid delivery model – offering both in-person and virtual consultations – demonstrated feasibility and adaptability, particularly for rural and remote populations. The study also incorporated a health economic evaluation, adding policy-relevant insights into the potential value of supportive care models. Furthermore, the intervention was grounded in existing clinical infrastructure, supporting its scalability and integration into routine care.

However, the study has some limitations that must be considered. Although secondary power calculations were conducted, the actual sample size achieved (*n* = 32) was lower than planned, limiting the study’s power. Moreover, attrition reduced the sample size, thereby widening the CI, although the effect size was substantial. Evaluating multiple outcomes within a modest sample may have impacted the precision of detecting significant differences. These limitations underscore the need for cautious interpretation and highlight the importance of future studies with larger samples to strengthen inference. The 90-day duration did not allow for evaluation of the durability or sustainability of the intervention. The reduction in unplanned presentations may be limited to the period of active intervention. Lastly, this model was implemented at 2 sites (1 rural and 1 urban) within the same state (NSW). This may limit the generalizability and scalability to other health systems with different patient profiles or different service structures, necessitating independent replication in larger or “different” cohorts. Therefore, several enabling factors, such as organizational preparedness, the availability of qualified personnel to implement the intervention components, the presence of pathways for integrating specialist and supportive care, and adequate funding to encourage proactive rather than reactive service delivery, are likely to be necessary for successful scale-up.

This paper presents an economic evaluation limited to a costing analysis of health service utilization. A more comprehensive evaluation was not feasible at this stage. However, a model-based cost–utility analysis, drawing on the findings of this study and existing literature, should be embedded in future research.

## Conclusion

In conclusion, this study provides preliminary evidence that the transdisciplinary model of care for ALD patients may improve their need for unplanned care through an early, coordinated, and value-based supportive approach. This prospective pilot RCT offers a foundation for future research, supporting ongoing studies of effectiveness, transferability, and sustainability of transdisciplinary supportive care models.

## Supporting information

10.1017/S1478951526101795.sm001 Pullen et al. supplementary material Pullen et al. supplementary material

## Supplementary material

The supplementary material for this article can be found at https://doi.org/10.1017/S1478951526101795.
