# Supplementary material for: Evaluating a transdisciplinary supportive care model for advanced liver disease: Results of the Liver Life pilot randomized controlled trial (RCT)
Source: Palliat Support Care. 2026 Feb 27;24:e74. doi: 10.1017/S1478951526101795 (PMC13166402; doi:10.1017/S1478951526101795)
Supplement: Pullen et al. supplementary material [file S1478951526101795sup001.docx]

**Supplementary files**

**Figure 1: Framework for a supportive care model**

**
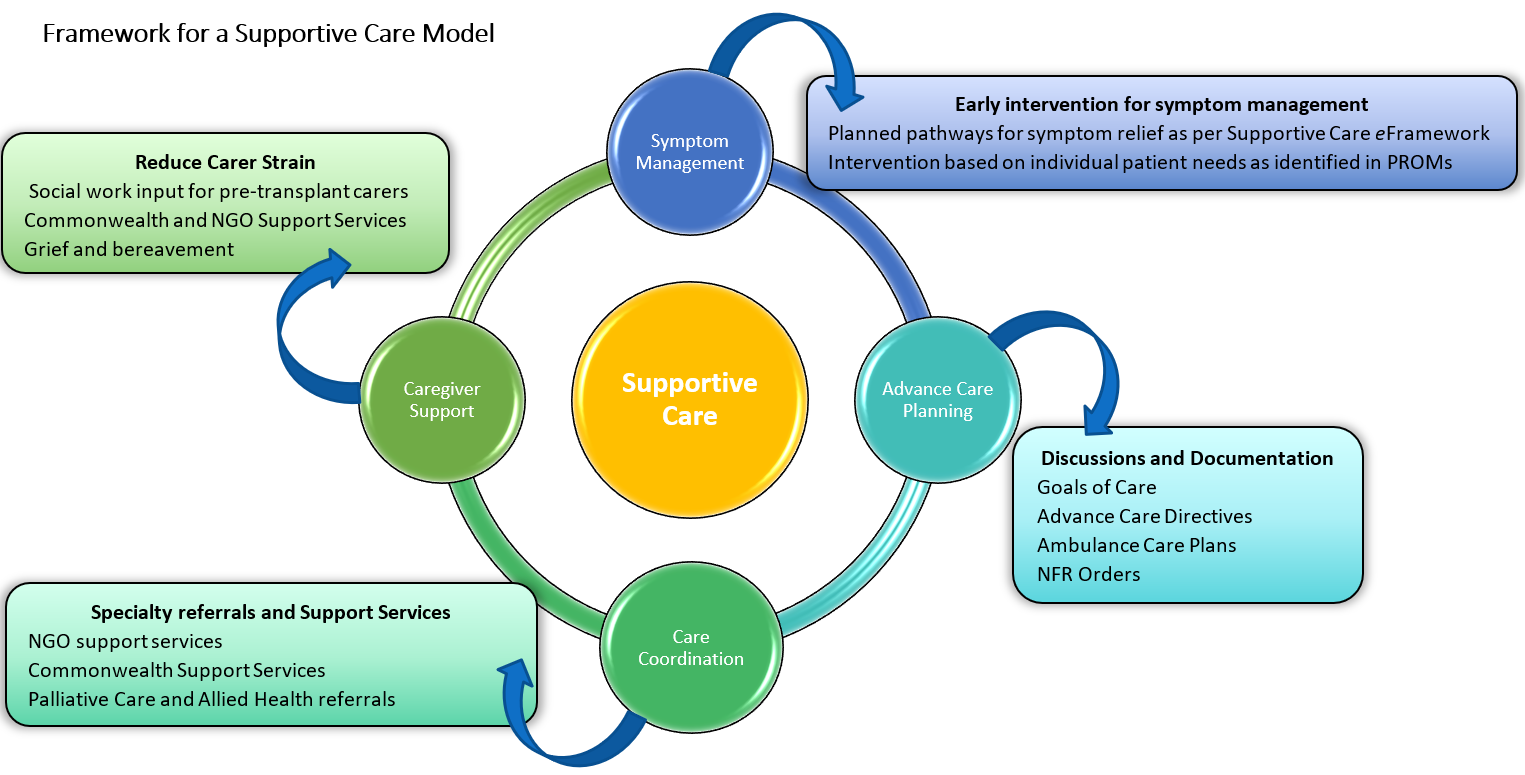
**

**Table 1: Triggers for intervention and the triggers activated across the 90-day trial.**

| **Patient/Carer Reported Measure** | **Item Number** | **Action** | **Number of occasions triggered** |
| --- | --- | --- | --- |
| Integrated Palliative Outcomes Scale (IPOS) | Q1 | Clinician reviewing survey to refer to appropriate clinician for intervention | 17 |
|  | Q2 | If score >2 for any item, refer for nurse or medical review for symptom management | 17 |
|  | Q3-8 | If score >2 for any item, refer for social work, nurse or medical review for anxiety/depression/wellbeing management | 17 |
|  | Q9 | If score >2 for any item, refer for social work or nurse review for psychosocial/wellbeing management. | 17 |
| Euroqol 5 Dimension 5 Level (EQ 5D 5L) | All items | If score moderate/severe/unable, refer for medical review +/- allied health assessment. If score no problem/slight, continue to monitor and consider escalation for medical review or allied health assessment as appropriate. | 12 |
| Malnutrition Screening Tool (MST) & Subjective Global Assessment (SGA) | Total score | If MST total score >2, refer to dietitian for nutritional assessment (SGA) and tailored intervention. | 12 |
| Carer Experience Scale (CES) | Any item | If score >2, refer for social work and/or nursing review for wellbeing management and care coordination | 7 |
| Carer Support Needs Assessment Tool (CSNAT) | Any item | If score a little more/quite a bit more/very much more, refer for social work and/or nursing review for care coordination. | 6 |

*Inclusive of participants who completed trial to week 12

**Table 2: Cost per visit to health services**

|  | **ED presentation**  **($)*** | **Hospital admission**  **($)*** | **Outpatient**  **($)*** |
| --- | --- | --- | --- |
| **Standard care**  (n = 15) | 1,101 | 10,077 | 324 |
| **Intervention**  (n = 17) | 1,185 | 6,316 | 324 |

*Rounded to whole numbers

**Table 3: QALYs for patient reported health related quality of life**

|  | **Mean** | **SD** | **Min** | **Max** |
| --- | --- | --- | --- | --- |
| **Standard care**  (n = 15) | 0.14 | 0.06 | 0.01 | 0.22 |
| **Intervention**  (n = 17) | 0.18 | 0.02 | 0.13 | 0.22 |

*Rounded to two decimal place
